# New Properties of a Bioinspired Pyridine Benzimidazole Compound as a Novel Differential Staining Agent for Endoplasmic Reticulum and Golgi Apparatus in Fluorescence Live Cell Imaging

**DOI:** 10.3389/fchem.2018.00345

**Published:** 2018-08-15

**Authors:** Felipe M. Llancalahuen, Juan A. Fuentes, Alexander Carreño, César Zúñiga, Dayán Páez-Hernández, Manuel Gacitúa, Rubén Polanco, Marcelo D. Preite, Ramiro Arratia-Pérez, Carolina Otero

**Affiliations:** ^1^Escuela de Química y Farmacia, Facultad de Medicina, Universidad Andres Bello, Santiago, Chile; ^2^Laboratorio de Patogénesis y Genética Bacteriana, Facultad de Ciencias de la Vida, Universidad Andres Bello, Santiago, Chile; ^3^Center of Applied Nanosciences, Universidad Andres Bello, Santiago, Chile; ^4^Fondo Nacional de Ciencia y Tecnología (FONDECYT), Santiago, Chile; ^5^Facultad de Química y Biología, USACH, Santiago, Chile; ^6^Centro de Biotecnología Vegeta, Facultad de Ciencias de la Vida, Universidad Andres Bello, Santiago, Chile; ^7^Departamento de Química Orgánica, Facultad de Química, Pontificia Universidad Católica de Chile, Santiago, Chile

**Keywords:** benzimidazole, fluorescence, hydrogen bond, differential staining, endoplasmic reticulum, Golgi apparatus

## Abstract

In this study, we explored new properties of the bioinspired pyridine benzimidazole compound **B2** (2,4-di-tert-butyl-6-(3H-imidazo[4,5-c]pyridine-2-yl)phenol) regarding its potential use as a differential biomarker. For that, we performed 1D ^1^HNMR (TOCSY), UV-Vis absorption spectra in different organic solvents, voltammetry profile (including a scan-rate study), and TD-DFT calculations that including NBO analyses, to provide valuable information about **B2** structure and luminescence. In our study, we found that the **B2** structure is highly stable, where the presence of an intramolecular hydrogen bond (IHB) seems to have a crucial role in the stability of luminescence, and its emission can be assigned as fluorescence. In fact, we found that the relatively large Stokes Shift observed for **B2** (around 175 nm) may be attributed to the stability of the **B2** geometry and the strength of its IHB. On the other hand, we determined that **B2** is biocompatible by cytotoxicity experiments in HeLa cells, an epithelial cell line. Furthermore, in cellular assays we found that **B2** could be internalized by passive diffusion in absence of artificial permeabilization at short incubation times (15 min to 30 min). Fluorescence microscopy studies confirmed that **B2** accumulates in the endoplasmic reticulum (ER) and Golgi apparatus, two organelles involved in the secretory pathway. Finally, we determined that **B2** exhibited no noticeable blinking or bleaching after 1 h of continuous exposure. Thus, **B2** provides a biocompatible, rapid, simple, and efficient way to fluorescently label particular organelles, producing similar results to that obtained with other well-established but more complex methods.

## Introduction

The capability to distinguish and identify different subcellular compartments is critical for understanding organelle function, biogenesis, and cell maintenance, as well as for describing protein sorting and intracellular trafficking pathways (Watson et al., 2005). To this end, cell imaging is becoming a powerful tool to reveal particular biological structures, and even molecular mechanisms, unraveling dynamics and functions of many cellular processes. Accordingly, the development of diverse transmitted light microscopy approaches, including fluorescence microscopy, is increasingly contributing to improve this technique. In this context, the research of new, improved fluorescent indicators clearly constitutes a challenge (Sanderson et al., 2014; Wollman et al., 2015). One of the most desired properties of the fluorophores is their ability to differentially interact with discrete structures in the cell; to this end, the understanding of the chemical properties of these fluorophores is crucial in the design of this kind of molecules. Nowadays, most intracellular compartments can be detected by specific labeling through fluorophores conjugated to particular molecules, such as antibodies, which provide differential binding. For instance, endoplasmic reticulum (ER) and Golgi apparatus, two organelles that work together in the secretory pathway and protein sorting of eukaryotic organisms, which can be stained with fluorophores conjugated to either anti-protein disulfide isomerase (PDI) or anti-58K mouse monoclonal antibody, respectively (Bielaszewska et al., 2013, 2017; Cañas et al., 2016). Nevertheless, the use of antibodies is complex because it requires several steps, remarking the need of alternatives, such as transfection of gene fusions. These gene fusions harbor domains that are differentially sorted inside the cell fused with luminescent domains that allow their identifications via fluorescence microscopy. Although extensively used, transfection is a technique that requires a couple of days to be performed (Kingston et al., 2001). On the other hand, differential dyes have been reported for to specifically stain cellular organelles. In this sense, the organic dye DiOC_6_(3) (3-hexyl-2-(3-(3-hexyl-2(3H)-benzoxazolylidene)-1-propenyl)-iodide), a lipophilic, cationic, green fluorescent compound, has been used to specifically stain the ER (Sabnis et al., 1997, 2009). In addition, fluorescent analogs of ceramide have proved to be especially valuable for specifically labeling the Golgi apparatus, which receives, processes, and sorts newly synthesized proteins exported from the ER (Cooper, 2000). Although these dyes readily accumulate in the ER and in Golgi apparatus of most cell types by a preferential membrane partitioning process, both stains present biocompatibility problems, thereby they must be functionalized with bovine serum albumin (BSA), prolonging and complicating the staining protocol (Sabnis et al., 1997).

Recently, we demonstrated that 2,4-di-tert-butyl-6-(3H-imidazo[4,5-c]pyridine-2-yl)phenol (**B2**), a neutral benzimidazole derivate exhibiting an intramolecular hydrogen bond (IHB). **B2** shows luminescent emission at room temperature, with a large Stokes shift (i.e., λ_ex_ = 335 nm; λ_em_ = 510 nm in acetonitrile) (Carreño et al., 2016). Furthermore, **B2** has proved to efficiently stain free bacteria (i.e., *Salmonella enterica* and *Escherichia coli*), biofilms of *Lactobacillus kunkei* and *L. rhamnosus*, and epithelial cell lines (SKOV-3 and HEK-293), as assessed by confocal microscopy (Carreño et al., 2016; Berríos et al., 2017). Interestingly, when epithelial cells were observed, we distinguished a punctuated pattern, apparently located in the cytoplasm. These results strongly suggest that **B2** is differentially staining a particular structure/organelle in those cells. Thus, in this work we explored the potential use of **B2** as a differential, antibody-free fluorophore in epithelial cells. To this purpose, we performed studies aimed to characterize its optical and electrochemical features, to better understand the role of the intramolecular hydrogen bond in the luminescent properties of **B2**. To support experimental findings, we also performed computational calculations using DFT theory. In addition, we found that **B2** is a biocompatible molecule that generates a punctuate pattern in an epithelial cell line (HeLa). Furthermore, we found that **B2** uptake can be detected at short incubation times, apparently by passive a diffusion mechanism. Finally, we found that **B2** provides a rapid (30 min), simple (no cell permeabilization is required), biocompatible, and efficient way to fluorescently label both ER and Golgi apparatus, producing similar results to that obtained with other well-established methods. We also provide evidence that **B2** is a good candidate to be used as a new, differential, antibody-free fluorophore for organelles belonging to the cell secretory pathway, in time-lapse experiments or short videos with continuous exposure, even at low temperatures.

## Materials and methods

All chemicals and solvents were purchased from Merck or Aldrich and used without further purification. All solvents were stored over appropriate molecular sieves prior to use.

### Synthesis of 2,4-di-tert-butyl-6-(3h-imidazo[4,5-C]pyridine-2-YL)phenol (B2)

The general procedure for the synthesis was previously reported, (Carreño et al., 2016) obtaining around 40% yield. Melting point: 311–312°C. FTIR (ATR, cm^−1^): 2961 (νOH), 2904 and 2868 (νNH), 1626 (νCe = N), 1526 (νC = C). ^1^HNMR (400 MHz, DMSO-_d6_, ppm): δ = 1.33 [s; 9H; tBu]; 1.43[s; 9H; tBu]; 7.40 [d, J = 1.9 Hz; 1H; H5], 7.69 [d; *J* = 4.7 Hz; 1H; H2], 8.02 [s; 1H; H4], 8.36 [d; J = 5.3 Hz; 1H; H1], 8.99 [s; 1H; H3], 13.60[s; 1H; O–H]. UV/VIS: (chloroform, room temperature) λ nm (ε mol^−1^ dm^3^ cm^−1^): 332 (13.32 × 10^3^), 294 (19.09 × 10^3^), 284 (15.51 × 10^3^); (acetonitrile, room temperature) λ nm (ε mol^−1^ dm^3^ cm^−1^): 327 (12.91 × 10^3^), 292 (18.67 × 10^3^), 282 (14.88 × 10^3^); (DMSO, room temperature) λ nm (ε mol^−1^ dm^3^ cm^−1^): 332 (11.30 × 10^3^), 294 (15.44 × 10^3^), 284 (12.23 × 10^3^). Rf: 0.38 (ethylacetate as solvent).

### Physical measurements

NMR spectra were recorded on a Bruker AVANCE 400 spectrometer operating at 400 MHz, at 25°C. Samples were dissolved in deuterated dimethyl sulfoxide (DMSO-_d6_), using tetramethylsilane as internal standard. FTIR techniques were recorded in an UATR spectrum Two Perkin Elmer spectrophotometer.

Purity of **B2** was checked by TLC using glass plates pre-coated with SiliaPlate TLC Aluminum foil TLC were supplied by Silicycle as stationary phase, and a suitable solvent system was used as mobile phase (ethyl acetate). Spots were visualized with short wave ultraviolet light (λ = 254 nm) using Spectroline LongLife TM Filter. Melting points were determined on a Stuart 10 Scientific melting point apparatus SMP3 (UK) in open capillary tubes.

For electrochemical experiments, a working solution containing 0.01 mol/L of the respective compound together with 0.1 mol/L tetrabutylammonium hexafluorophosphate (TBAPF_6_) as supporting electrolyte in CH_3_CN, was used. Prior to each experiment, the working solution was purged with high purity argon, and an argon atmosphere was maintained during the whole experiment. A polycrystalline non-annealed platinum disc (2 mm diameter) was used as working electrode. A platinum gauze of large geometrical area, separated from the cell main compartment by a fine sintered glass, was used as counter electrode. All potentials quoted in this work were referred to an Ag/AgCl electrode in tetramethylammonium chloride to match the potential of a saturated calomel electrode (SCE), at room temperature. All electrochemical experiments were performed at room temperature on a CHI900B bipotentiostat interfaced to a PC running the CHI 9.12 software that allowed experimental control and data acquisition.

### ^1^H-NMR and Ftir characterization of B2

^1^H-NMR and FTIR techniques were performed as previously described for **B2** (Carreño et al., 2016). These techniques were used to confirm the correct synthesis of **B2**.

### Computational details

All structural and electronic properties were obtained using the Amsterdam Density Functional (ADF) code (Te Velde et al., 2001). All molecular structures were fully optimized by an analytical energy gradient method as implemented by Verluis and Ziegler (Echeverria et al., 2009; Ramírez-Tagle et al., 2010; Alvarado-Soto and Ramirez-Tagle, 2015; Bjorgaard et al., 2015), using the hybrid B3LYP functional and the standard Slater-type-orbital (STO) basis set with triple-ζ quality double plus polarization functions (TZ2P) for all the atoms (Rabanal-Leon et al., 2014; Zhang et al., 2016). Frequency analyses were performed after the geometry optimization to corroborate the minimum and to compare with experimental infrared spectra. Natural bond orbital (NBO) analysis was used to characterize energies of the IHB (Avilés-Moreno et al., 2017; Guajardo Maturana et al., 2017). Time-dependent density functional theory (TDDFT) (Ghane et al., 2012; Fuks et al., 2013; Mosquera and Wasserman, 2015), used at the same level of theory to calculate the excitation energies using in all cases the conductor-like screening model for realistic solvents (COSMO) (Sinnecker et al., 2006; Simpson et al., 2015; Yamin et al., 2016), DMSO to estimate the hydrogen bond stability and to visualize the conformational changes due to the solvent polarity, additionally the calculations were also performed in the gas phase (Tsolakidis and Kaxiras, 2005; Quartarolo and Russo, 2011).

### Cell culture

The HeLa cell line (ATCC® CCL-2™) (cervical adenocarcinoma) was grown in 25 cm^2^ polystyrene bottles in Dulbecco's High Glucose Modified Eagle Medium (DMEM) supplemented with 10% v/v fetal serum bovine (FBS), 1 mM sodium pyruvate and 1% v/v penicillin-streptomycin. Cells were incubated at 37°C and 5% CO_2_, changing the culture medium every 2–3 days, and propagated when they reached between 80 and 90% confluence.

### Cellular staining

HeLa cells were seeded in a 24-well culture dish (3 × 10^5^ cells per well) in which a 12-mm diameter coverslip was previously added, and allowed to acclimate for 24 h. Each well was then washed 3 times with sterile 1 × PBS and then the different concentrations of **B2** (200, 100, 50, 25, or 12.5 μg/mL) and DMSO vehicle (50, 25, 12.5, 6.125, and 3.0625%) were added to each well and incubated for 15 and 30 min at 37°C with 5% CO_2_. It is important to underline that permeabilization procedures are not necessary. Subsequently, each well was washed 3 times with sterile 1 × PBS and coverslips were deposited on the slides using 5 μl of Fluoromount® mounting medium. Each slide was left to dry in the dark at room temperature overnight and were sealed with acrylic paint.

### Fluorescence microscopy

The analysis was performed on a Model BX61 Fluorescence Microscope (Olympus Corp., Tokyo, Japan), Spinning Disk Olympus (DSU) system, coupled to an ORCA-R2 camera (Hamamatsu Photonics KK, Japan) and CellSens Dimension software v1.9 (Olympus Corp., Tokyo, Japan). Fluorescence emission was obtained by excitation with a xenon lamp. Emission was collected with a DAPI filter (450 to 500 nm).

### B2 cellular uptake

HeLa cells (2 × 10^5^ cells/well) were seeded in 24-well plates and incubated for 24 h. To evaluate the cellular uptake of compound **B2**, concentrations of 25 μg/mL and 50 μg/mL were prepared in culture medium, added to each well and incubated for 15 and 30 min. In addition, to evaluate whether the uptake of **B2** depends on energy, cells were incubated with the same treatments at 4°C, a temperature where no endocytic processes occur (Mukherjee et al., 1997).

After the incubation time, cells were washed 3 times with 1 × PBS and peeled off the culture plate using a 0.2% w/v PBS-EDTA solution and incubated for 20 min at RT. Suspended cells were washed twice with FACS buffer (2% PBS supplemented with fetal bovine serum) and resuspended in 500 μl PBS. Finally, the fluorescence intensity of the cells was quantified through a BD Accuri™ FACSARIA II flow cytometer using the FlowJo 7.6.1 software.

### Cell viability assays

HeLa cells were cultured in Dulbecco's Modified Eagle's Medium (DMEM) containing 10% fetal bovine serum (FBS), 2 mM L-glutamine, 100 units/mL penicillin and 100 μg/mL streptomycin. Cells were maintained in 75 cm^2^ flasks in a 5% CO_2_-humidified atmosphere at 37°C. Passage took place every 2–3 days. All cell culture supplies were purchased from Sigma-Aldrich. Toxicity was determined using the 3-(4,5-dimethylthiazol-2-yl)-2,5- diphenyltetrazolium bromide (MTT) cell viability assay after 15 min, 30 min, 1 h, and 24 h of incubation with **B2**. MTT is a yellow compound that, when reduced by active mitochondria, produces purple formazan crystals that can be measured spectrophotometrically (Low et al., 2016; Sheikh et al., 2016). For this purpose, MTT (Sigma-Aldrich) was dissolved in phosphate buffered saline (PBS) to a concentration of 5 mg/mL and further diluted in culture medium (1:11). Cells were incubated with this MTT-solution for 4 h under normal culture conditions. Afterwards, 100 μL of isopropyl alcohol were added. To completely dissolve the formazan salts, plates were incubated for 10 min on a shaker and quantified by measuring absorbance at 570 nm with an ELISA microplate reader. Cell viability was calculated as percentage of surviving cells compared to untreated control cells.

### Transformation of chemically competent TOP-10 *Escherichia coli*

For propagation of the desired vector, it was introduced into the chemically competent *E. coli* TOP10 (ThermoFisher)^1^ (generated according to the manufacturer's instructions). The transformation was performed using 100 μL of the chemo-competent bacteria and 1 μg of circular vector (KDEL-GFP or sialyl transferase signal anchor sequence-RFP). The mixture was incubated on ice for 30 min and a thermal shock was quickly performed at 42°C for 2 min, immediately after, was put on ice for 3 min and 900 μL of LB broth was added and incubated for 1 h at 37°C. Finally, the bacteria were plated on LB agar supplemented with 50 μg/mL kanamycin for the selection of the transforming colonies.

### Cellular transfection

HeLa cells were seeded in a 24-well culture dish (5 × 10^5^ cells per well) in which a glass cover was previously added to each well and allowed to acclimate for 24 h. Subsequently, the entire culture medium was removed, and each well was washed 3 times with sterile 1 × PBS. The different combinations of DNA and Lipofectamine 3000® Transfection Reagent were performed according to the manufacturer's instructions. After all components were added to the cell culture (Lipofectamine 3000® Transfection Reagent, DNA and DMEM medium), it was incubated for 6 h at 37°C with 5% CO_2_. The content of each well was removed and washed 3 times with 1 × sterile PBS. Then, 500 μL of complete DMEM medium was added to each well and incubated until 48 h of treatment were completed. Finally, cell transfection was checked using an inverted-light microscope and a BX-53 epifluorescence microscope.

### Co-localization assays

HeLa cells were seeded in a 24-well culture dish with 5 × 10^5^ cells per well in which a 12-mm diameter coverslip was previously added and allowed to acclimate for 24 h. Cells were then transfected with the KDEL-GFP and Sialyl-RFP plasmids, which express specific fluorescent peptide of the endoplasmic reticulum and Golgi apparatus, respectively. After the transfection process was completed, each well was washed 3 times with sterile 1 × PBS and then the different concentrations of **B2** (25 μg/mL and 50 μg/mL) and the percentages of vehicle DMSO (12.5 and 6.125%) were added for 15 min with 5% CO_2_ and at 37°C. Each well was then washed 3 times with sterile 1 × PBS and fixed for 5 min with 4% PFA in PBS (4g PFA, 1 M CaCl_2_, 1 M MgCl_2_, pH 7.4 adjusted solution), washed 3 times with PBS and each coverslip was mounted on slides using 5 μl of Electron Microscopy Sciences. Each slide was allowed to dry in the dark at room temperature overnight and then sealed with acrylic paint. Finally, all samples were observed using the Olympus BX-61-DSU epifluorescence microscope.

### Statistical analysis

All values of analyzed data are presented as mean standard error (SE) from three biological replicates. Statistical analysis included was one-way ANOVA followed by multiple comparison test (Tukey). Differences among groups were considered statistically significant when *p* < 0.05.

## Results and discussion

### B2 characterization

**B2** (Figure S1, see Table S1 for characteristic constants) is insoluble in water, but presents low solubility in chloroform, acetonitrile and methanol, and a good solubility in DMSO at room temperature. **B2** synthesis was confirmed by their FTIR (Figures S2, S3), ^1^H-NMR spectra (for proton numbering, see Figure S4; for ^1^H-NMR see Figure S5) including TOCSY experiments (Figure S6–S8).

Since **B2** is being tested as a new fluorophore for biological applications, its use in different solvents may be also desirable. In this context, electronic absorption spectra of **B2** were measured in different organic solvents: chloroform, acetonitrile, and DMSO, at room temperature. We observed three intense absorption bands. The first two high-intensity absorption can be assigned to n → π^*^ (–C = N–) and π → π^*^ transitions, respectively. No significant shifts were observed for **B2** in the different solvents used (see Table S2), suggesting that the IHB in **B2** is stable under all the tested conditions, even in presence of DMSO, which can form hydrogen bonds with the solute. Any change in the **B2** structure (including dissociation of the IHB), produced by interaction with the solvent, would lead to changes in the absorption spectra. This was not observed for **B2** under the tested conditions (see Figure S9 for UV-Vis spectrum).

To complement the previously reported electrochemical characterization of **B2** (Carreño et al., 2016), a scan-rate study was performed at 50, 200, and 400 mVs^−1^. We found that **B2** exhibited a single reversible reduction process [${\text{Red}}_{\left(\text{rev}\right)}^{\text{I}}$] at −0.84 V, and two irreversible oxidations, ${\text{Ox}}_{\left(\text{irr}\right)}^{\text{I}}$ and ${\text{Ox}}_{\left(\text{irr}\right)}^{\text{II}}$, at 0.96 and 1.47 V vs. SCE (saturated calomel electrode), respectively, consistent to previously reported data (Carreño et al., 2016). As inferred from the Figure S10, the control mechanism for all the studied processes depends on the species diffusion from the bulk solution (see Table S3). Diffusional control of red-ox processes has been reported for similar benzimidazoles and other bioinspired compounds (Savarino et al., 1997; Boiani et al., 2006; Moore et al., 2008; Manbeck et al., 2016), but the reduction in those cases has been found to be irreversible. The difference is that **B2** possesses an IHB that has been attributed to stabilize the radical form, explaining the reversibility of reduction and emphasizing the importance of the IHB in the **B2** features (Benisvy et al., 2006; Moore et al., 2010).

### Theoretical calculations

**Figure 1:**
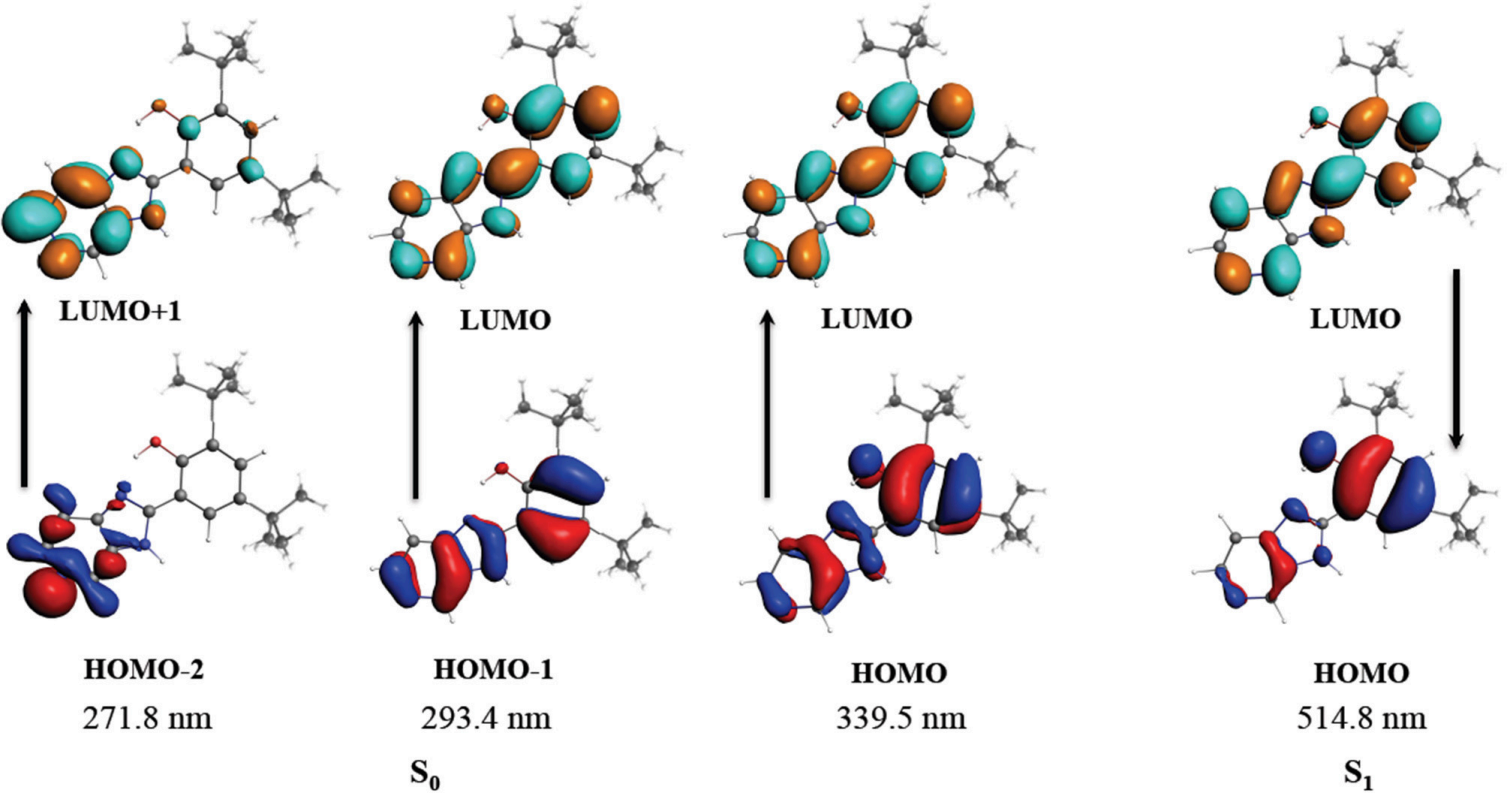
Molecular orbitals involved in the absorption and emission for **B2**. S_0_ corresponds to the ground state. S_1_ corresponds to the first excited singlet state.

As stated, **B2** is a luminescent compound (Carreño et al., 2016). To better characterize this phenomenon, we performed time-dependent density functional theory (TDDFT) calculations to assign the electronic transitions. The first step was to optimize the ground (S_0_) and the first excited singlet state (S_1_); in a second step, absorption and emission bands were calculated using TDDFT. All the calculations where performed using COSMO model for solvent with the parameters of DMSO (Liu et al., 2011). We found that the geometry of S_0_ and S_1_ exhibited no significant differences, showing, in both cases, that the structure must remain planar, most likely due to the presence of the IHB (see Table S4). To further study the **B2** IHB, we evaluated the second-order interaction energy by calculating natural bond orbitals (NBO). We found that the IHB energy is 6.23 kcal/mol for S_0_, and 6.13 kcal/mol for S_1_. These values are in agreement with the reported values in similar compounds harboring IHB (Muhammad et al., 2010; Abdel Ghani and Mansour, 2012; Monajjemi, 2012; Carreño et al., 2016; Yankova and Radev, 2016), and support the stability of this interaction (Sosa et al., 2002). In this sense, the experimental UV-vis results (see Table S2) were corroborated by computational methods (see and Figure S9). To further characterize the UV-vis observed transitions, TDDFT calculations were conducted (see Table S5). This calculated transition is composed of a HOMO-2 → LUMO+1 (n → π^*^), HOMO-1 → LUMO (π → π^*^), and HOMO → LUMO (π → π^*^). The band located experimentally at 332 nm (DMSO), theoretically calculated at 339 nm, corresponded to a HOMO → LUMO transition. Both the HOMO and LUMO composition involves the IHB (Figure 1). The isosurfaces provide some suggestions regarding experimental results obtained from UV-Vis studies (see Table S2 and Figure S9). All these results together demonstrate the stability of the IHB in **B2**.

On the other hand, the emission band was calculated using the geometry of the first excited state S_1_ to perform a TDDFT calculation. The emission was calculated as a π^*^ → π between LUMO and HOMO orbitals at 515 nm, in good agreement with the experimental value reported around 500 nm (data not shown) (Carreño et al., 2016). As stated above, HOMO and LUMO composition involves the IHB, reinforcing the contribution of this interaction in the stability of **B2**. The rigidity of the **B2** structure substantially reduces the vibronic relaxation, explaining the luminescence of this compound, as reported for other molecules (Gopal et al., 1995). Regarding the luminescence of **B2**, the geometry of the first excited triplet was also calculated and used for a TDDFT calculation using a previously described protocol (Carreño et al., 2017). However the contribution of the triplet to the emission band was less than 0.5%, indicating that the emission can be assigned to fluorescence, as previously suggested (Carreño et al., 2016). Altogether, the combined information obtained by the analyses described above, provides valuable information about **B2** structure, and its luminescence.

### Cellular studies

**Figure 2:**
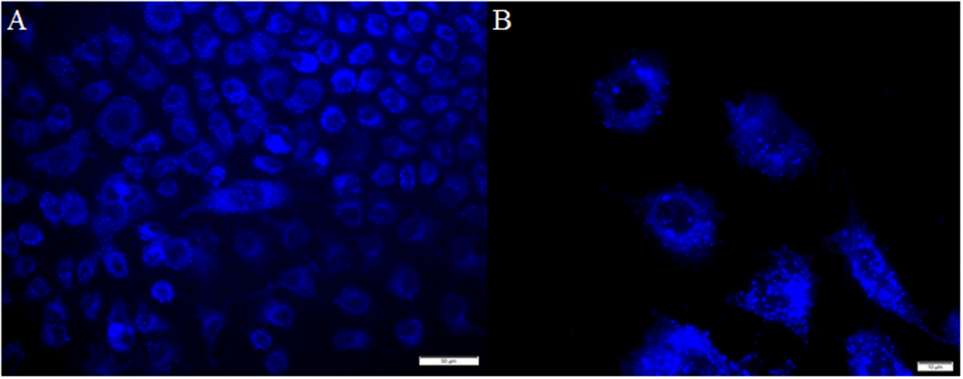
Labeling pattern of **B2**. HeLa cells were seeded on coverslips and incubated with 50 μg/mL **B2** for 30 min at 37°C prior being fixed and analyzed by fluorescence microscopy. Bar size 50 μm **(A)** and 10 μm **(B)**. We found similar results with a shorter (15 min at 37°C) incubation and/or with 25 μg/mL **B2**; albeit not staining was observed with 12.5 μg/mL **B2** (data not shown).

To further characterize **B2** in cellular assays, we stained HeLa cells (epithelial cell line) with **B2** (200, 100, 50, 25, or 12.5 μg/mL) for 30 min, prior to fluorescent microscopy. Beforehand, we characterized the **B2** staining properties using a confocal microscope, with laser excitation at 405 nm, and emission collected with a long-pass filter in the range of 425 to 525 nm (Carreño et al., 2016). Although confocal microscopy exhibits several advantages, such as the possibility to examine samples through the *Z* axis, it needs special requirements. For that reason, this time we used a epifluorescence microscope, using a xenon lamp (excitation) and DAPI filter (emission, 450 to 500 nm) (Atale et al., 2014). We found that **B2** showed a suitable fluorescence inside cells, were the optimal concentration ranged between 25 and 50 μg/mL of **B2** (Figure 2). When 12.5 μg/mL **B2** was used, we were unable to observe fluorescence under the tested conditions (data not shown). Interestingly, we observed that **B2** produced a punctuate pattern inside cells (Figure 2), strongly suggesting that **B2** is not a general, but a differential stain. In addition, it is important to remark that, albeit 30 min of incubation with **B2** are optimal, 15 min are sufficient to visualize cells under the fluorescence microscope (data not shown), highlighting the potential of this compound as a relatively quick biomarker, even using common equipment, such as a xenon or mercury lamp, and a DAPI filter. Moreover, it is clear from these experiments that chemical derivatizations are not required for **B2** to be used as biomarker. Finally, the **B2** staining protocol can be performed without the need of additional permeabilization steps, underlining its simplicity. In this sense, DMSO has been known to enhance cell membrane permeability of drugs or DNA. Studies exploring DMSO applicability to promote plasma membrane permeability using different molecules in living cells, demonstrated that DMSO can increase cell permeabilization, in a both concentration- and time-depending manner (de Ménorval et al., 2012). It has been reported that 10% v/v of DMSO slightly increase cell permeability after 1 h of incubation, but its impact in the entry of polar molecules is marginal, as assessed by the absence of swelling and the limited amount of water that could cross the plasma membrane. Although the presence of small undulations in the plasma membrane were reported in eukaryotic cells treated with 10% DMSO, they were only visible after 1 h in the presence of DMSO (de Ménorval et al., 2012). Considering that be used 25 or 50 μg/ml of **B2** to stain cells (involving the presence of 6.25 and 12.5% v/v DMSO, respectively), and shorter times of incubation (i.e., 15 or 30 min), we speculate that the DMSO could contribute to the entry of **B2** into epithelial cells, with minimal effects on cell morphology. Nevertheless, the potential impact of the DMSO in cellular compartments, at the concentrations and incubation times proposed in this study for **B2** staining protocol, must be explored in future analyses.

### B2 cellular uptake

**Figure 3:**
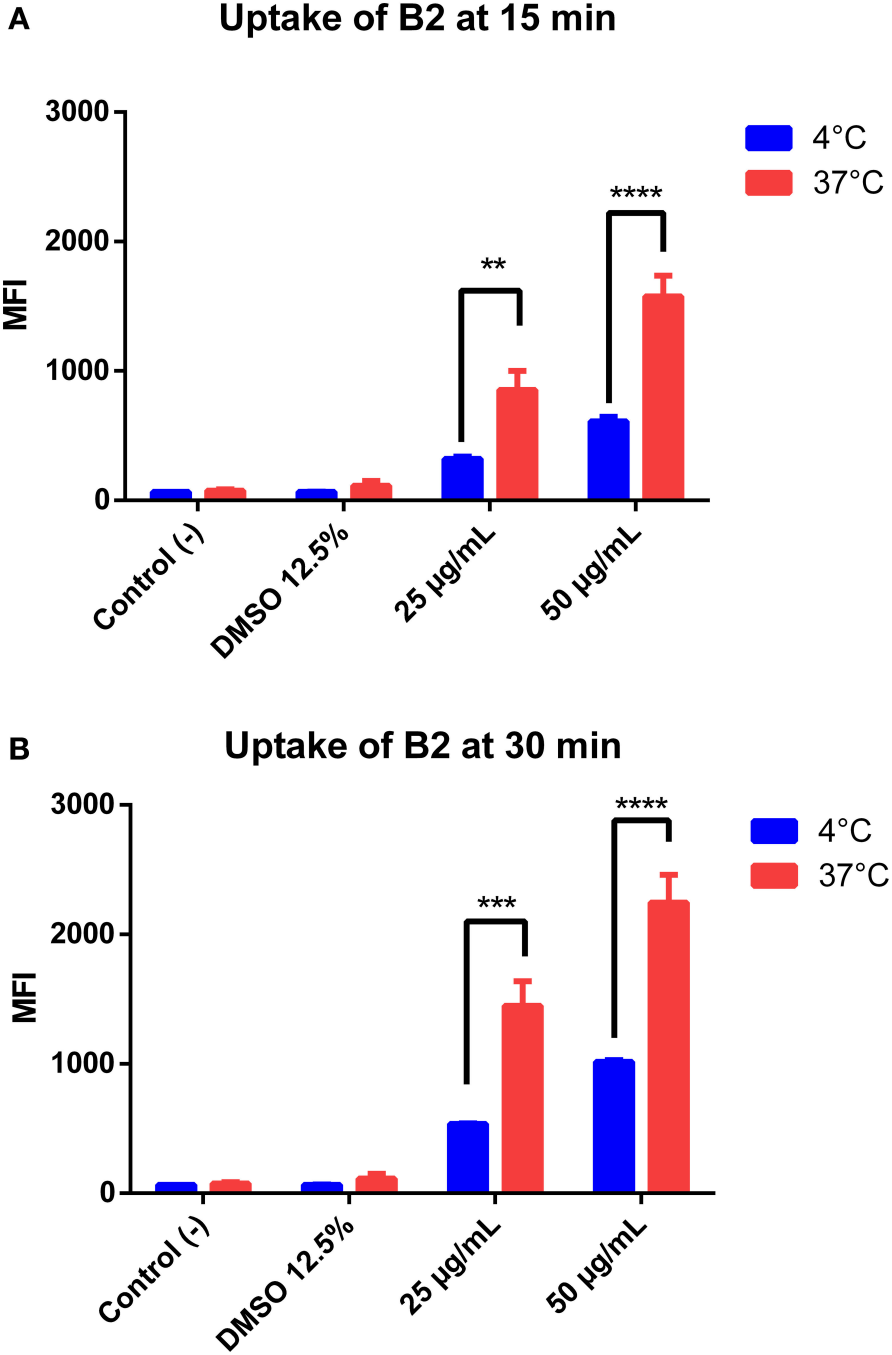
Quantification of **B2** uptake into HeLa cells. HeLa cells were incubated with different concentrations of **B2** (25 or 50 μg/mL), at 4°C or 37°C, for 15 min **(A)** or 30 min **(B)**. The mean fluorescence intensity (MFI) was determined by flow cytometry using a 350-nm excitation laser and a 520-nm detector. The statistical difference is based on the negative control (culture medium alone). A 2-way ANOVA was performed with Tukey's posttest as a statistical analysis. Only relevant differences are depicted in the figure. **p < 0.01, ***p < 0.001, ****p < 0.0001. *n* = 3 (biological triplicate).

To better understand the **B2** potential as biomarker, we characterized its cellular uptake in epithelial cells. For that, we performed flow cytometry assays of cells stained under three different conditions: (1) Two **B2** concentrations that allow cell staining (25 and 50 μg/mL); (2) two incubation times with **B2**: 15 min, suboptimal for staining, and 30 min, optimal for staining; and (3) two temperatures (4 and 37°C). We determined its cellular uptake under the last condition because it is particularly valuable to assess the entry mechanism, since active endocytic processes are inhibited at 4°C (Mukherjee et al., 1997). As shown in Figure 3, **B2** cellular uptake is dependent on both incubation time and concentration. Although **B2** uptake significantly decreases at 4°C compared to the uptake observed at 37°C, cells still showed considerable **B2**-dependent luminescence at 4°C, especially when cells were incubated for 30 min. In fact, the decreased uptake at 4°C can be explained by the diminished plasma membrane fluidity, since at low temperatures the diffusion rate is impaired (Cooper and Sunderland, 2000). Since active uptake is inhibited at 4°C, we infer that **B2** is internalized by cells through passive diffusion. This property explains why **B2** staining protocol does not require a permeabilization step, providing a simplified staining method. Other authors reported Ir (III)-based (*d*^6^) compounds as potential biomarkers that were unable to enter cells at 4°C, even after prolonged incubation periods (more than 2 h) (Yin Zhang et al., 2010; Zhang et al., 2010). This fact remarks the **B2** advantages in comparison with other fluorophores, even *d*^6^ complexes, regarding cell labeling. Thus, some of the advantages exhibited by **B2** include its use at low temperatures and low incubation times, valuable properties if considered that not additional permeabilization steps are required.

In general, factors determining the entry of fluorophores into cells include size, charge, and hydrophobicity (Juris et al., 1988; Stufkens, 1998; Puckett and Barton, 2007; Yin Zhang et al., 2010; Zhao et al., 2011; Gill and Thomas, 2012). Thus, **B2** might permeate inside cells due to its own chemical nature, by the contribution of the DMSO as an incorporated permeabilizer agent in **B2** solutions, or by a combination of this features. Nevertheless, since the DMSO contributes to a slight plasma membrane permeabilization only after 1 h of incubation at the concentrations used for staining (de Ménorval et al., 2012), we cannot rule out that **B2**, itself, is able to penetrate cells by passive transport.

All these properties must be considered to design efficient chemical compounds as cellular biomarkers. Another desirable property for a biomarker is the differential staining. Differential staining is the ability to specifically stain a particular cell structure (e.g., organelles such as endoplasmic reticulum or Golgi apparatus). Normally, biomarkers can be modified to be used as differential dye through conjugation with antibodies, relatively big and complex molecules that usually preclude cell uptake; thereby, additional permeabilization steps are required. By contrast, **B2** provides a simpler method to achieve differential staining without the need of antibodies, as we will discuss below.

### B2 is suitable as cellular biomarker

**Figure 4:**
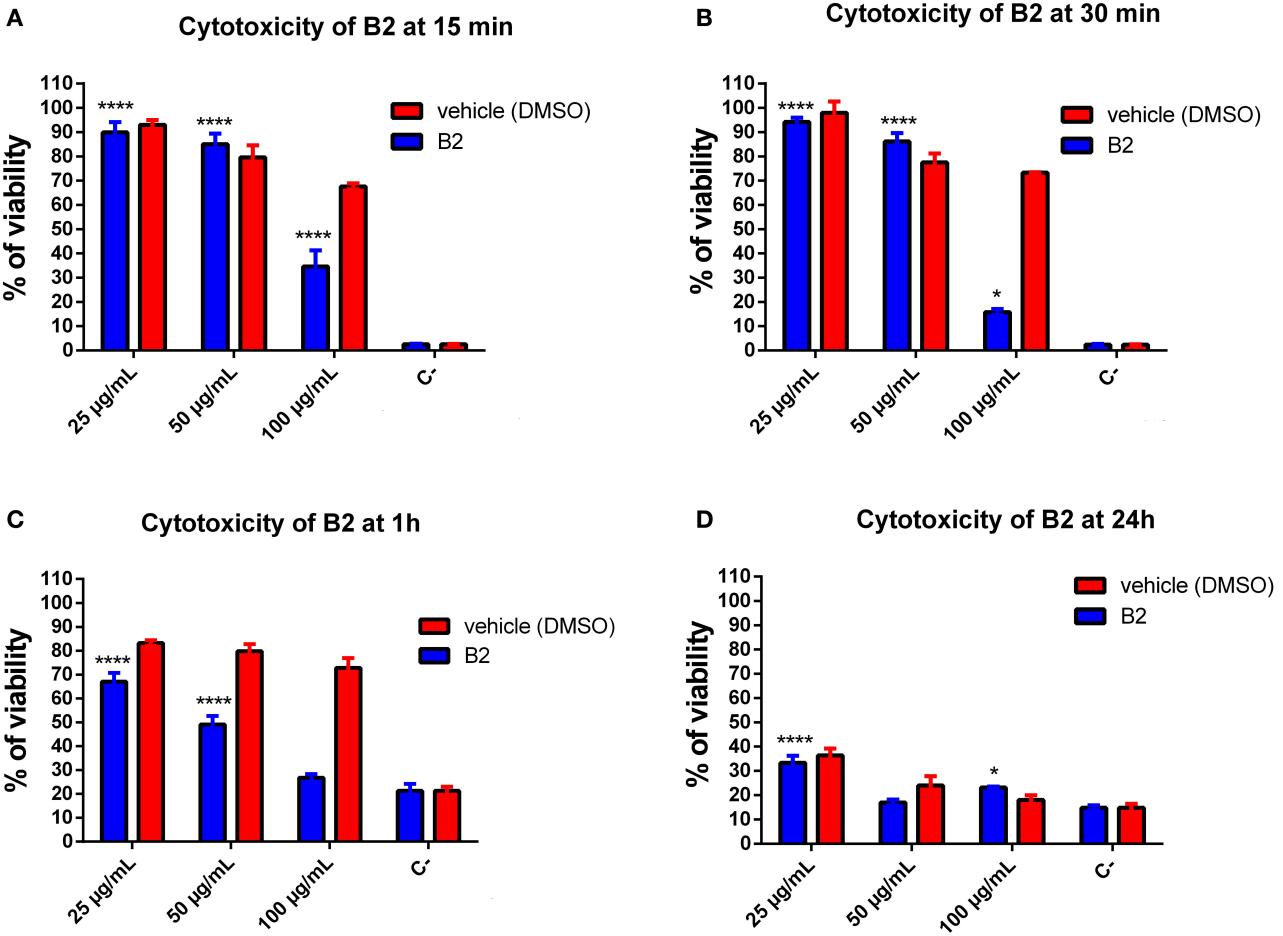
Quantification of **B2** cytotoxicity in HeLa cells. Cytotoxicity assay was performed using the MTT Cell Growth Kit. HeLa cells were incubated with **B2** (25 μg/mL or 50 μg/mL) for 15 **(A)**, 30 min **(B)**, 1 **(C)**, and 24 h **(D)** at 37°C. Percentages are corrected based on the viability control (culture medium alone). The significant difference is based on the negative control (DMSO 100%). A 2-way ANOVA was performed with Tukey's posttest as a statistical analysis. Only relevant differences are depicted in the figure. *p < 0.05, ****p < 0.0001. *n* = 3 (biological triplicate).

A suitable fluorophore for live cell imaging should exhibit three main properties: good and stable luminescence, efficient cellular uptake, and low cytotoxicity (Haas and Franz, 2009). In this context, we evaluated **B2** cytotoxicity in epithelial cells by MTT assays. MTT is a yellow compound that, when reduced by functioning mitochondria, produces purple formazan crystals that can be measured spectrophotometrically (Low et al., 2016; Sheikh et al., 2016). We tested different concentrations of **B2** (25, 50, and 100 μg/mL) and different incubation times (15 min, 30 min, 1 h, and 24 h). It is important to remark that the optimal staining protocol requires 50 μg/mL of **B2**, and 30 min of incubation (Figure 2). Figures 4A,B show that, at the staining conditions, **B2** presented low cytotoxicity (around 10%) compared with the vehicle alone (i.e., DMSO), values that represent no toxicity for cellular models (Nel et al., 2009). By contrast, more prolonged incubation times, or higher concentrations, produced more pronounced effects (Figures 4C,D). In these cases, cytotoxicity increased by approximately 33% at 1 h incubation time using 25 μg/mL, and at 52% using 50 μg/mL, which can be due to an excessive accumulation of **B2** in organelles and to the presence of DMSO (vehicle). DMSO increases cyclic adenosine monophosphate (cAMP), a second messenger involved in cell death by apoptosis (Cho et al., 2014). These results indicate that **B2** can be used in living systems as biomarker at relatively short incubation times (15 to 30 min), whereas more prolonged times (i.e., >1 h) are not recommended due to higher cytotoxicity levels, not biocompatible for these kind of studies (i.e., >20%) (Nel et al., 2009). These results show that **B2** exhibited low cytotoxicity under the staining conditions proposed in this work, proving its biocompatibility as biomarker.

### B2 accumulates in endoplasmic reticulum and golgi apparatus

As shown in Figure 2, **B2** presented a punctuate staining pattern, with a central unstained area plausibly corresponding to the cell nucleus. This strongly suggests that **B2** is accumulated in a discrete, particular cell structure (e.g., an organelle). It has been reported that endoplasmic reticulum (ER) exhibit a similar staining pattern (Li et al., 2016). The ER and Golgi apparatus are two organelles that work together in the secretory pathway of eukaryotic proteins (Hanada, 2017). Thus, we proposed that **B2** is being accumulated in these organelles. To test this hypothesis, we performed co-localization experiments using known intracellular fluorescent markers normally used as reference: KDEL-GFP [lysine/aspartate/glutamate/leucine–green fluorescent protein (emission: 503–508 nm)], to stain the ER; and Sialyl-RFP [Sialyl transferase signal anchor sequence—red fluorescent protein (583 nm)], to stain the Golgi apparatus (Tsien, 1998; Dayel et al., 1999; Remington, 2002; Hawes and Satiat-Jeunemaitre, 2005). These reference biomarkers, i.e., KDEL-GFP and Sialyl-RFP, are recombinant fluorescent proteins that accumulates in secretory organelles. As described above, the staining protocol of **B2** is simple, consisting mainly in short incubation times (30 min). Unlike **B2**, KDEL-GFP and Sialyl-RFP need to be expressed directly by the cells, thereby a transfection protocol must be performed. Transfection consists in introducing purified nucleic acids, normally produced in bacteria, into eukaryotic cells to express heterologous proteins, such as KDEL-GFP or sialyl-RFP. Complete transfection protocol can take two or more days.

**Figure 5:**
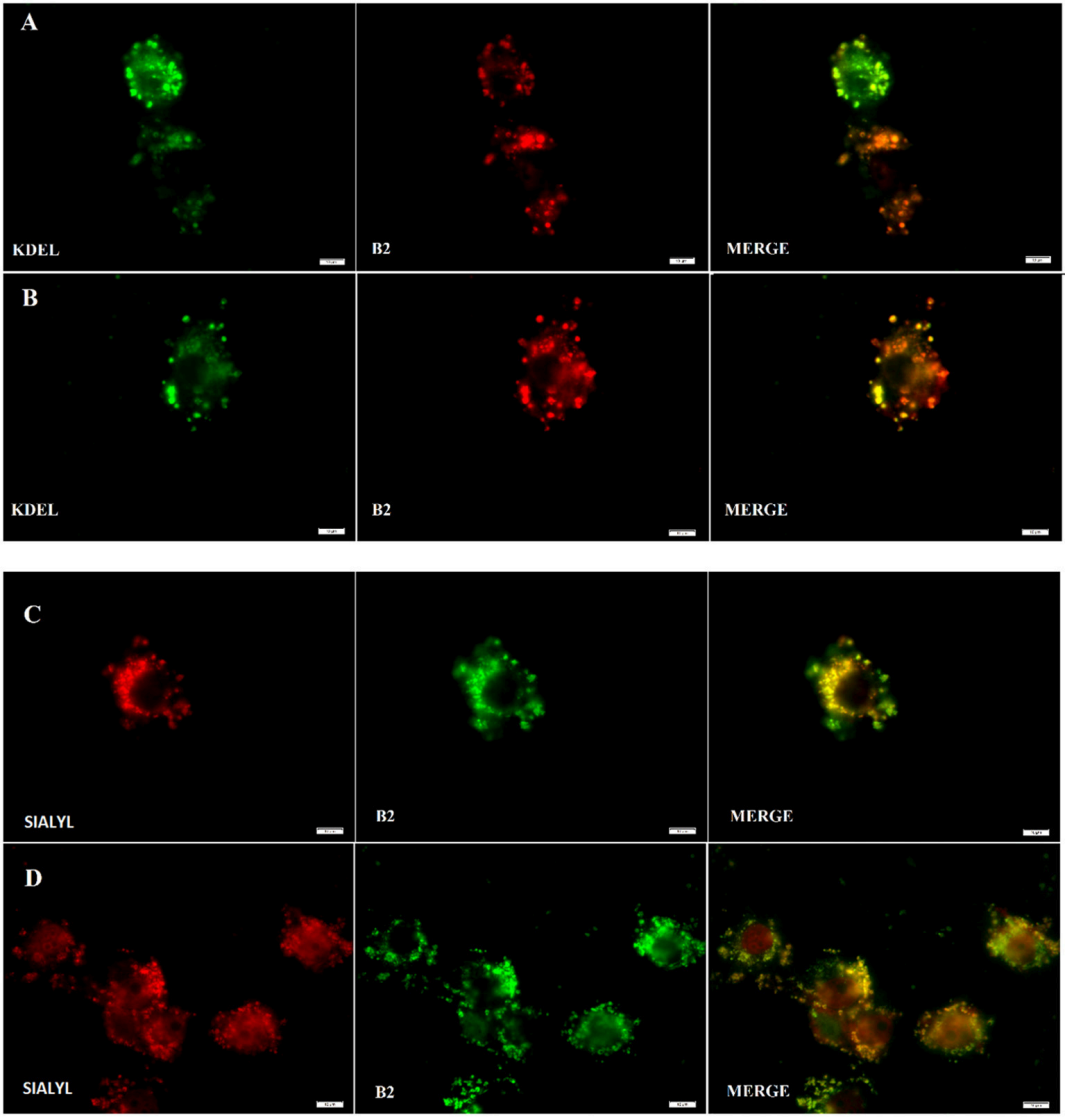
**B2** staining co-localizes with Endoplasmic Reticulum (ER) **(A,B)** and Golgi apparatus **(C)**, **(D)**. Fluorescence assay was performed using HeLa cells and analyzed under fluorescent microscopy at 48 h post-transfection either with KDEL-GFP gene **(A,B)** or with Sialyl-RFP **(C,D)**. To stain with **B2**, respective transfected cells were simply incubated with **B2** at 50 μg/mL for 30 min at 37°C. In the case of **B2**, a pseudo color was used to facilitate its visualization and co localization. White bars represent 10 μm.

As shown in Figure 5, **B2** accumulates in subcellular compartments from the secretory pathways (i.e., ER and Golgi apparatus), revealing these organelles as efficiently as KDEL-GFP or Sialyl-RFP, but with a simpler method. **B2** accumulation in the ER and in the Golgi apparatus is probably due to its affinity for certain proteins inside these organelles, probably glycoproteins. Zhang et al. demonstrated that two fluorophores based on Ir (III) present high affinity for the Golgi apparatus (Zhang et al., 2010). These Ir (III)-based compounds possess many pyridine groups, which are also present in the **B2** structure. Nevertheless, comparing **B2** and Ir (III)-based fluorophores, **B2** is a simpler molecule exhibiting better quantum yields (φ = 0.21 in acetonitrile) (Carreño et al., 2016), whereas Ir (III)-based fluorophores are complex dendritic cyclometalated compounds exhibiting lower quantum yields (ϕ = 0.036 to 0.14 in acetonitrile, depending on the compound). Most importantly, visualization of Golgi apparatus in HeLa cells requires 2 h of incubation with 2 M of Ir (III) complexes to obtain similar results (Zhang et al., 2010) to those shown in the Figure 5, which needed lower incubation time at lower concentration (155 μM) of **B2**. Furthermore, although other fluorophores have been reported to stain the ER, such as the ER-tracker (C_44_H_42_BClF_2_N_6_O_7_S_2_), (ThermoFisher^1^; Diwu et al., 1997) their considerable size mandatorily requires cell permeabilization, a step that is not necessary in the case of the **B2** staining protocol.

On the other hand, it has been reported that DiOC_6_(3) is able to stain the ER at 10 μg/mL. Nevertheless, at lower concentrations (i.e., 0.1 μg/mL), DiOC_6_(3) stains mitochondria instead, letting unclear which are the limit concentrations to stain one organelle or another (Sabnis et al., 2009). By contrast, **B2** stain ER and Golgi at 50 μg/mL (or more), whereas lower concentrations appear to be unable to stain (as mentioned above), allowing a better identification.

Thus, unlike other fluorophores which only have cytoplasmic or perinuclear distribution (Puckett and Barton, 2007; Yin Zhang et al., 2010; Zhang et al., 2010), **B2** provides a simple and efficient way to differentially stain ER and Golgi apparatus in epithelial cells, showing that **B2** can be considered a differential fluorescent dye.

### Blinking and bleaching B2 properties

**Figure 6:**
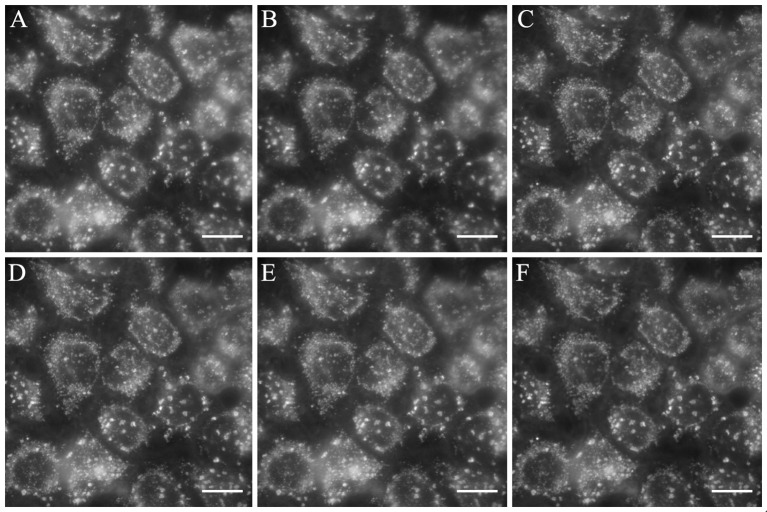
**B2** exhibits resistance to photobleaching. HeLa cells were stained with 50 μg/mL **B2** for 30 min at 37°C and observed under the fluorescent microscope immediately **(A)**, or 5 min **(B)**, 10 min **(C)**, 15 min **(D)**, 30 min **(E)**, 60 min **(F)** after the staining protocol in continuous exposure to the exciting light. We obtained similar results with MCF-7 cells (data not shown). Blinking was not observed (see Supplementary Video 1). White bar represents 10 μm.

Some fluorophores exhibit unpredictable blinking properties, a clear drawback to obtain high quality images (Michalet et al., 2005; Mahler et al., 2008). On the other hand, photobleaching is also an undesired property of fluorophores when time-lapse experiments or videos under continuous exposure are required (Li et al., 2017; Elisa et al., 2018). For instance, DiOC_6_(3) exhibits photobleaching similar to that of the rhodamine (Sabnis et al., 2009). To test whether **B2** exhibits photobleaching and/or blinking in biological applications, we stained HeLa cells with 50 μg/mL **B2** for 30 min at 37°C. Then, stained cells were observed by fluorescence microscopy during 1 h with continuous exposure. As shown in Figure 6, **B2** is resistant to photobleaching, and blinking was not observed (see Supplementary Video 1). This phenomenon can be explained by the high stability of **B2**, as demonstrated above. The IHB, that is stable in different organic solvents (Table S2), contributes to keep the rigidity between the benzimidazole and phenolic ring moieties, substantially reducing the vibronic relaxation due to a coplanar geometry between the rings, and contributing to the fluorescence by minimizing the non-radiative emission. All this evidence suggests that, at least in part, the presence of a stable IHB contributes to strongly decrease photobleaching of **B2**.

## Conclusions

In this work, we explored new features of the luminescent compound **B2** concerning some of its chemical properties, and its use as biomarker for specific cell organelles. We found that **B2** exhibits a very stable structure, a feature that in turn contributes to a photobleaching-resistant fluorescence. In addition, **B2** provides a rapid (30 min for optimal staining), simple (since no cell permeabilization is required), biocompatible (low cytotoxicity under staining conditions), and efficient way to fluorescently label both ER and Golgi apparatus, producing similar results to that obtained with other well-established methods, but without photobleaching or blinking. Altogether, our results show that **B2** is suitable to be used for differential labeling of ER and Golgi apparatus, in time-lapse experiments or short videos with continuous exposure, even at low temperatures.

## Dedication

Dedicated to Professor Juan Manuel Manríquez on the occasion of his retirement.

## Author contributions

FL: Cytotoxicity and uptake experiment, discussion of biological experiment and paper writing; JF: Discussion of all the experiments, and paper writing; AC: Synthesis, characterization, discussion of all the experiments, and paper writing; CZ: Optical and other chemical characterizations; DP-H: Theoretical calculations; MG: Electrochemical studies; RP: Discussion of Biological experiments; MP: Discussion of NMR, TOCSY and FTIR experiments; RA-P: Discussion of theoretical calculations; CO: Microscopy and discussion of Biological experiments.

### Conflict of interest statement

The authors declare that the research was conducted in the absence of any commercial or financial relationships that could be construed as a potential conflict of interest.

## Footnotes

^1^*Thermofisher ER-Tracker*™ *Red (BODIPY*™ *TR Glibenclamide), for Live-Cell Imaging [Online]*. Available online at: http://www.thermofisher.com/order/catalog/product/E34250 [Accessed].

## Supplementary material

Figure S1 Molecular model of pyridine benzimidazole 2,4-di-*tert*-butyl-6-(3H-imidazo[4,5-c]pyridine-2-yl)phenol (**B2**) used in this study; front **(A)** and slide **(B)**.

Figure S2 FTIR (ATR) of precursor (phenyl-3,5-di-tert-butyl-2-hydroxybenzoate).

Figure S3 FTIR (ATR) of **B2**.

Figure S4 Numbering protons of **B2**.

Figure S5 Aromatic zone of the ^1^HNMR spectrum of **B2**.

Figure S6 1D TOCSY of **B2** (in DMSO-_d6_), irradiating H3 at 3,597 Hz (8.99 ppm).

Figure S7 1D TOCSY of **B2** (in DMSO-_d6_), irradiating H4 at 3,208 Hz (8.02 ppm).

Figure S8 1D TOCSY of **B2** (in DMSO-_d6_), irradiating -OH at 5,451 Hz (13.62 ppm).

Figure S9 UV-Vis spectrum of **B2** in chloroform, acetonitrile and DMSO at room temperature.

Figure S10 Scan-rate study for **B2**. Interphase: Pt|1.0·10^−2^ M **B2** + 1.0·10^−1^ M TBAPF_6_ in anhydrous CH_3_CN.

Table S1 Characteristic constants of phenyl-3,5-di-tert-butyl-2-hydroxybenzoate^*^ and **B2**.

Table S2 UV-Vis absorption spectra of **B2** in different organic solvents at room temperature.

Table S3 Scan-rate study for determining diffusional control of described electrochemical processes at **B2**.

Table S4 Results for the geometry optimization of the ground (S0) and first excited singlet (S1) state.

Table S5 The most important absorption and emission bands for B2 in DMSO.

Supplementary Video 1 HeLa cells were seeded on coverslips and incubated with 50 μg/mL of B2 for 30 min at 37°C. Then, cells were observed under fluorescent microscope with constant exposure of 350 msec.

## References

[B1] Abdel Ghani N. T. Mansour A. M. (2012). Molecular structures of 2-arylaminomethyl-1H-benzimidazole: spectral, electrochemical, DFT and biological studies. Spectrochim Acta A Mol. Biomol. Spectrosc. 91, 272–284. 10.1016/j.saa.2012.01.080 22381803

[B2] Alvarado-Soto L. Ramirez-Tagle R. (2015). A theoretical study of the binding of [Re_6_Se_8_(OH)_2_(H_2_O)_4_] rhenium clusters to DNA purine base guanine. Materials 8, 3938–3944. 10.3390/ma8073938 28793416 PMC5455628

[B3] Atale N. Gupta S. Yadav U. C. Rani V. (2014). Cell-death assessment by fluorescent and nonfluorescent cytosolic and nuclear staining techniques. J. Microsc. 255, 7–19. 10.1111/jmi.12133 24831993

[B4] Avilés-Moreno J. R. Berden G. Oomens J. Martinez-Haya B. (2017). Isolated complexes of the amino acid arginine with polyether and polyamine macrocycles, the role of proton transfer. Phys. Chem. Chem. Phys. 19, 31345–31351. 10.1039/c7cp04270a 29149235

[B5] Benisvy L. Bill E. Blake A. J. Collison D. Davies E. S. Garner C. D. (2006). Phenoxyl radicals: H-bonded and coordinated to Cu(II) and Zn(II). Dalton Trans. 2006, 258–267. 10.1039/b513221p 16357984

[B6] Berríos P. Fuentes J. A. Salas D. Carreno A. Aldea P. Fernandez F.. (2017). Inhibitory effect of biofilm-forming *Lactobacillus kunkeei* strains against virulent *Pseudomonas aeruginosa in vitro* and in honeycomb moth (*Galleria mellonella*) infection model. Benef. Microbes 9, 257–268. 10.3920/BM2017.0048 29124967

[B7] Bielaszewska M. Rüter C. Bauwens A. Greune L. Jarosch K. A. Steil D.. (2017). Host cell interactions of outer membrane vesicle-associated virulence factors of enterohemorrhagic *Escherichia coli* O157: intracellular delivery, trafficking and mechanisms of cell injury. PLoS Pathog. 13:e1006159. 10.1371/journal.ppat.1006159 28158302 PMC5310930

[B8] Bielaszewska M. Rüter C. Kunsmann L. Greune L. Bauwens A. Zhang W.. (2013). Enterohemorrhagic *Escherichia coli* hemolysin employs outer membrane vesicles to target mitochondria and cause endothelial and epithelial apoptosis. PLoS Pathog. 9:e1003797. 10.1371/journal.ppat.1003797 24348251 PMC3861543

[B9] Bjorgaard J. A. Velizhanin K. A. Tretiak S. (2015). Solvent effects in time-dependent self-consistent field methods. II. Variational formulations and analytical gradients. J. Chem. Phys. 143:054305. 10.1063/1.4927167 26254651

[B10] Boiani M. Boiani L. Denicola A. Torres De Ortiz S. Serna E. Vera De Bilbao N.. (2006). 2H-benzimidazole 1,3-dioxide derivatives: a new family of water-soluble anti-trypanosomatid agents. J. Med. Chem. 49, 3215–3224. 10.1021/jm0600343 16722639

[B11] Cañas M. A. Giménez R. Fábrega M. J. Toloza L. aldomà L. Badia J. (2016). Outer membrane vesicles from the probiotic *Escherichia coli* Nissle 1917 and the commensal ECOR12 enter intestinal epithelial cells via clathrin-dependent endocytosis and elicit differential effects on DNA damage. PLoS ONE 11:e0160374. 10.1371/journal.pone.0160374 27487076 PMC4972321

[B12] Carreño A. Gacitúa M. Fuentes J. A. Páez-Hernández D. Araneda C. Chávez I. (2016). Theoretical and experimental characterization of a novel pyridine benzimidazole: suitability for fluorescence staining in cells and antimicrobial properties. New J. Chem. 40, 2362–2375. 10.1039/C5NJ02772A

[B13] Carreño A. Solis-Céspedes E. Páez-Hernández D. Arratia-Pérez R. (2017). Exploring the geometrical and optical properties of neutral rhenium (I) tricarbonyl complex of 1,10-phenanthroline-5,6-diol using relativistic methods. Chem. Phys. Lett. 685, 354–362. 10.1016/j.cplett.2017.07.058

[B14] Cho E. A. Kim E. J. Kwak S. J. Juhnn Y. S. (2014). cAMP signaling inhibits radiation-induced ATM phosphorylation leading to the augmentation of apoptosis in human lung cancer cells. Mol. Cancer 13:36. 10.1186/1476-4598-13-36 24568192 PMC4234305

[B15] Cooper G. M. (2000). The Cell: A Molecular Approach. Boston, MA: Boston University.

[B16] Cooper G. M. Sunderland M. A. (2000). The Cell: A Molecular Approach. Boston, MA.

[B17] Dayel M. J. Hom E. F. Verkman A. S. (1999). Diffusion of green fluorescent protein in the aqueous-phase lumen of endoplasmic reticulum. Biophys. J. 76, 2843–2851. 10233100 10.1016/S0006-3495(99)77438-2 PMC1300255

[B18] de Ménorval M. A. Mir L. M. Fernandez M. L. Reigada R. (2012). Effects of dimethyl sulfoxide in cholesterol-containing lipid membranes: a comparative study of experiments *in silico* and with cells. PLoS ONE 7:e41733. 10.1371/journal.pone.0041733 22848583 PMC3404987

[B19] Diwu Z. Lu Y. Zhang C. Klaubert D. H. Haugland R. P. (1997). Fluorescent molecular probes II. The synthesis, spectral properties and use of fluorescent solvatochromic dapoxyl dyes. Photochem. Photobiol. 66, 424–431.

[B20] Echeverria C. Santibanez J. F. Donoso-Tauda O. Escobar C. A. Ramirez-Tagle R. (2009). Structural antitumoral activity relationships of synthetic chalcones. Int. J. Mol. Sci. 10, 221–231. 10.3390/ijms10010221 19333443 PMC2662465

[B21] Elisa Z. Toon B. De Smedt S. C. Katrien R. Kristiaan N. Kevin B. (2018). Technical implementations of light sheet microscopy. Microsc. Res. Tech. 10.1002/jemt.22981 [Epub ahead of print]. 29322581

[B22] Fuks J. I. Elliott P. Rubio A. Maitra N. T. (2013). Dynamics of charge-transfer processes with time-dependent density functional theory. J. Phys. Chem. Lett. 4, 735–739. 10.1021/jz302099f 26281927

[B23] Ghane T. Brancolini G. Varsano D. Di Felice R. (2012). Optical properties of triplex DNA from time-dependent density functional theory. J. Phys. Chem. B 116, 10693–10702. 10.1021/jp304818s 22866829

[B24] Gill M. R. Thomas J. A. (2012). Ruthenium(II) polypyridyl complexes and DNA–from structural probes to cellular imaging and therapeutics. Chem. Soc. Rev. 41, 3179–3192. 10.1039/c2cs15299a 22314926

[B25] Gopal V. R. Reddy A. M. Rao V. J. (1995). Wavelength-dependent trans to cis and quantum chain isomerizations of anthrylethylene derivatives. J. Organ. Chem. 60, 7966–7973.

[B26] Guajardo Maturana R. Valenzuela M. L. Schott E. Rojas-Poblete M. (2017). Bonding and optical properties of spirocyclic-phosphazene derivatives. A DFT approach. Phys. Chem. Chem. Phys. 19, 31479–31486. 10.1039/c7cp06064e 29159340

[B27] Haas K. L. Franz K. J. (2009). Application of metal coordination chemistry to explore and manipulate cell biology. Chem. Rev. 109, 4921–4960. 10.1021/cr900134a 19715312 PMC2761982

[B28] Hanada K. (2017). Ceramide transport from the endoplasmic reticulum to the trans golgi region at organelle membrane contact sites. Adv. Exp. Med. Biol. 997, 69–81. 10.1007/978-981-10-4567-7_5 28815522

[B29] Hawes C. Satiat-Jeunemaitre B. (2005). The plant Golgi apparatus–going with the flow. Biochim. Biophys. Acta 1744, 93–107. 10.1016/j.bbamcr.2005.03.009 15922463

[B30] Juris A. Balzani V. Barigelletti F. Campagna S. Belser P. Von Zelewsky A. (1988). Ru(II) polypyridine complexes: photophysics, photochemistry, eletrochemistry, and chemiluminescence. Coord. Chem. Rev. 84, 85–277.

[B31] Kingston R. E. Chen C. A. Okayama H. (2001). Calcium phosphate transfection. Curr. Protoc. Immunol. 31, 1013.1–10.13.9. 10.1002/0471142735.im1013s31 18432676

[B32] Li J. F. Li C. Y. Aroca R. F. (2017). Plasmon-enhanced fluorescence spectroscopy. Chem. Soc. Rev. 46, 3962–3979. 10.1039/C7CS00169J 28639669

[B33] Li K. T. Chen Q. Wang D. W. Duan Q. Q. Tian S. He J. W.. (2016). Mitochondrial pathway and endoplasmic reticulum stress participate in the photosensitizing effectiveness of AE-PDT in MG63 cells. Cancer Med. 5, 3186–3193. 10.1002/cam4.895 27700017 PMC5119974

[B34] Liu Y. F. Yang D. P. Shi D. H. Sun J. F. (2011). A TD-DFT study on the hydrogen bonding of three esculetin complexes in electronically excited states: strengthening and weakening. J. Comput. Chem. 32, 3475–3484. 10.1002/jcc.21932 21919018

[B35] Low M. L. Maigre L. Tahir M. I. Tiekink E. R. Dorlet P. Guillot R.. (2016). New insight into the structural, electrochemical and biological aspects of macroacyclic Cu(II) complexes derived from S-substituted dithiocarbazate schiff bases. Eur. J. Med. Chem. 120, 1–12. 10.1016/j.ejmech.2016.04.027 27183379

[B36] Mahler B. Spinicelli P. Buil S. Quelin X. Hermier J. P. Dubertret B. (2008). Towards non-blinking colloidal quantum dots. Nat. Mater. 7, 659–664. 10.1038/nmat2222 18568030

[B37] Manbeck G. F. Fujita E. Concepcion J. J. (2016). Proton-coupled electron transfer in a strongly coupled photosystem ii-inspired chromophore-imidazole-phenol complex: stepwise oxidation and concerted reduction. J. Am. Chem. Soc. 138, 11536–11549. 10.1021/jacs.6b03506 27538049

[B38] Michalet X. Pinaud F. F. Bentolila L. A. Tsay J. M. Doose S. Li J. J.. (2005). Quantum dots for live cells, *in vivo* imaging, and diagnostics. Science 307, 538–544. 10.1126/science.1104274 15681376 PMC1201471

[B39] Monajjemi M. (2012). NMR and NBO calculation of benzimidazoles and pyrimidines: nano physical parameters investigation. Int. J. Phys. Sci. 7, 2010–2031. 10.5897/IJPS11.507

[B40] Moore G. F. Hambourger M. Gervaldo M. Poluektov O. G. Rajh T. Gust D.. (2008). A bioinspired construct that mimics the proton coupled electron transfer between P680^*^+ and the Tyr(Z)-His190 pair of photosystem II. J. Am. Chem. Soc. 130, 10466–10467. 10.1021/ja803015m 18642819

[B41] Moore G. F. Hambourger M. Kodis G. Michl W. Gust D. Moore T. A.. (2010). Effects of protonation state on a tyrosine-histidine bioinspired redox mediator. J. Phys. Chem. B 114, 14450–14457. 10.1021/jp101592m 20476732

[B42] Mosquera M. A. Wasserman A. (2015). Time-dependent electronic populations in fragment-based time-dependent density functional theory. J. Chem. Theory Comput. 11, 3530–3536. 10.1021/acs.jctc.5b00342 26574438

[B43] Muhammad S. Xu H. Janjua M. R. Su Z. Nadeem M. (2010). Quantum chemical study of benzimidazole derivatives to tune the second-order nonlinear optical molecular switching by proton abstraction. Phys. Chem. Chem. Phys. 12, 4791–4799. 10.1039/B924241D 20428560

[B44] Mukherjee S. Ghosh R. N. Maxfield F. R. (1997). Endocytosis. Physiol. Rev. 77, 759–803. 9234965 10.1152/physrev.1997.77.3.759

[B45] Nel A. E. Mädler L. Velegol D. Xia T. Hoek E. M. Somasundaran P.. (2009). Understanding biophysicochemical interactions at the nano-bio interface. Nat. Mater. 8, 543–557. 10.1038/nmat2442 19525947

[B46] Puckett C. A. Barton J. K. (2007). Methods to explore cellular uptake of ruthenium complexes. J. Am. Chem. Soc. 129, 46–47. 10.1021/ja0677564 17199281 PMC2747593

[B47] Quartarolo A. D. Russo N. (2011). A computational study (TDDFT and RICC2) of the electronic spectra of pyranoanthocyanins in the gas phase and solution. J. Chem. Theory. Comput. 7, 1073–1081. 10.1021/ct2000974 26606355

[B48] Rabanal-Leon W. A. Murillo-Lopez J. A. Paez-Hernandez D. Arratia-Perez R. (2014). Understanding the influence of terminal ligands on the electronic structure and bonding nature in [Re6(mu3-Q8)](2+) clusters. J. Phys. Chem. A 118, 11083–11089. 10.1021/jp508892r 25347816

[B49] Ramírez-Tagle R. Alvarado-Soto L. Hernández-Acevedo L. Arratia-Pérez R. (2010). Spin-orbit and solvent effects in the luminescent [Re6Q8(NCS)6]4-, Q = S, se, Te clusters: molecular sensors and molecular devices. J. Chilean Chem. Soc. 55, 39–43. 10.4067/s0717-97072010000100010

[B50] Remington S. J. (2002). Negotiating the speed bumps to fluorescence. Nat. Biotechnol. 20, 28–29. 10.1038/nbt0102-28 11753356

[B51] Sabnis R. W. Deligeorgiev T. G. Jachak M. N. Dalvi T. S. (1997). DiOC6(3): a useful dye for staining the endoplasmic reticulum. Biotech. Histochem. 72, 253–258. 9408585 10.3109/10520299709082249

[B52] Sabnis R. W. Deligeorgiev T. G. Jachak M. N. Dalvi T. S. (2009). DiOC6(3): a useful dye for staining the endoplasmic reticulum. Biotech. Histochem. 72, 253–258. 10.3109/10520299709082249 9408585

[B53] Sanderson M. J. Smith I. Parker I. Bootman M. D. (2014). Fluorescence microscopy. Cold Spring Harb. Protoc. (2014):pdb top071795. 10.1101/pdb.top071795 PMC4711767 25275114

[B54] Savarino P. Viscardi G. Quagliotto P. Perracino P. Barni E. (1997). Voltammetric behaviour of heterocyclic systems. Pyridyl-substituted benzimidazoles, benzoxazoles and benzothiazoles.1. J. Heterocyclic Chem. 34, 1479–1485.

[B55] Sheikh R. A. Wani M. Y. Shreaz S. Hashmi A. A. (2016). Synthesis, characterization and biological screening of some Schiff base macrocyclic ligand based transition metal complexes as antifungal agents. Arabian J. Chem. 9, S743–S751. 10.1016/j.arabjc.2011.08.003

[B56] Simpson S. Gross M. S. Olson J. R. Zurek E. Aga D. S. (2015). Identification of polybrominated diphenyl ether metabolites based on calculated boiling points from COSMO-RS, experimental retention times, and mass spectral fragmentation patterns. Anal. Chem. 87, 2299–2305. 10.1021/ac504107b 25565148

[B57] Sinnecker S. Rajendran A. Klamt A. Diedenhofen M. Neese F. (2006). Calculation of solvent shifts on electronic g-tensors with the conductor-like screening model (COSMO) and its self-consistent generalization to real solvents (direct COSMO-RS). J. Phys. Chem. A 110, 2235–2245. 10.1021/jp056016z 16466261

[B58] Sosa G. L. Peruchena N. M. Contreras R. H. Castro E. A. (2002). Topological and NBO analysis of hydrogen bonding interactions involving C–H…O bonds. J. Mol. Struc. 577, 219–228. 10.1016/s0166-1280(01)00670-4

[B59] Stufkens D. (1998). Ligand-dependent excited state behaviour of Re(I) and Ru(II) carbonyl–diimine complexes. Coord. Chem. Rev. 177, 127–179.

[B60] Te Velde G. Bickelhaupt F. M. Baerends E. J. Fonseca Guerra C. Van Gisbergen S. J. A. Snijders J. G. (2001). Chemistry with ADF. J. Comput. Chem. 22, 931–967. 10.1002/jcc.1056

[B61] Tsien R. Y. (1998). The green fluorescent protein. Annu. Rev. Biochem. 67, 509–544. 9759496 10.1146/annurev.biochem.67.1.509

[B62] Tsolakidis A. Kaxiras E. (2005). A TDDFT study of the optical response of DNA bases, base pairs, and their tautomers in the gas phase. J. Phys. Chem. A 109, 2373–2380. 10.1021/jp044729w 16839008

[B63] Watson P. Jones A. T. Stephens D. J. (2005). Intracellular trafficking pathways and drug delivery: fluorescence imaging of living and fixed cells. Adv. Drug Deliv. Rev. 57, 43–61. 10.1016/j.addr.2004.05.003 15518920

[B64] Wollman A. J. Nudd R. Hedlund E. G. Leake M. C. (2015). From Animaculum to single molecules: 300 years of the light microscope. Open Biol. 5:150019. 10.1098/rsob.150019 25924631 PMC4422127

[B65] Yamin P. Isele-Holder R. Leonhard K. (2016). Predicting octanol/water partition coefficients of alcohol ethoxylate surfactants using a combination of molecular dynamics and the conductor-like screening model for realistic solvents. Ind. Eng. Chem. Res. 55, 4782–4789. 10.1021/acs.iecr.5b04955

[B66] Yankova R. Radev L. (2016). Structural and electronic properties of [Co(benzimidazole)_2_I_2_]. Int. J. Mater. Chem. 6, 19–27. 10.5923/j.ijmc.20160602.01

[B67] Yin Zhang K. Law W. Lo K. K. (2010). Cyclometalated iridium(III) bipyridine complexes functionalized with an n-methylamino-oxy group as novel phosphorescent labeling reagents for reducing sugars. Organometallics 29, 3474–3476. 10.1021/om100597g

[B68] Zhang D. Zhao L. Zhu Y. Li A. He C. Yu H.. (2016). Effects of p-(Trifluoromethoxy)benzyl and p-(Trifluoromethoxy)phenyl molecular architecture on the performance of naphthalene tetracarboxylic diimide-based air-stable n-type semiconductors. ACS Appl. Mater. Interfaces 8, 18277–18283. 10.1021/acsami.6b04753 27355858

[B69] Zhang K. Y. Liu H. W. Fong T. T. Chen X. G. Lo K. K. (2010). Luminescent dendritic cyclometalated iridium(III) polypyridine complexes: synthesis, emission behavior, and biological properties. Inorg. Chem. 49, 5432–5443. 10.1021/ic902443e 20491455

[B70] Zhao Q. Huang C. Li F. (2011). Phosphorescent heavy-metal complexes for bioimaging. Chem. Soc. Rev. 40, 2508–2524. 10.1039/c0cs00114g 21253643

