# Supplementary figures and images for: New Properties of a Bioinspired Pyridine Benzimidazole Compound as a Novel Differential Staining Agent for Endoplasmic Reticulum and Golgi Apparatus in Fluorescence Live Cell Imaging
Source: Front Chem. 2018 Aug 15;6:345. doi: 10.3389/fchem.2018.00345 (PMC6123694; doi:10.3389/fchem.2018.00345)

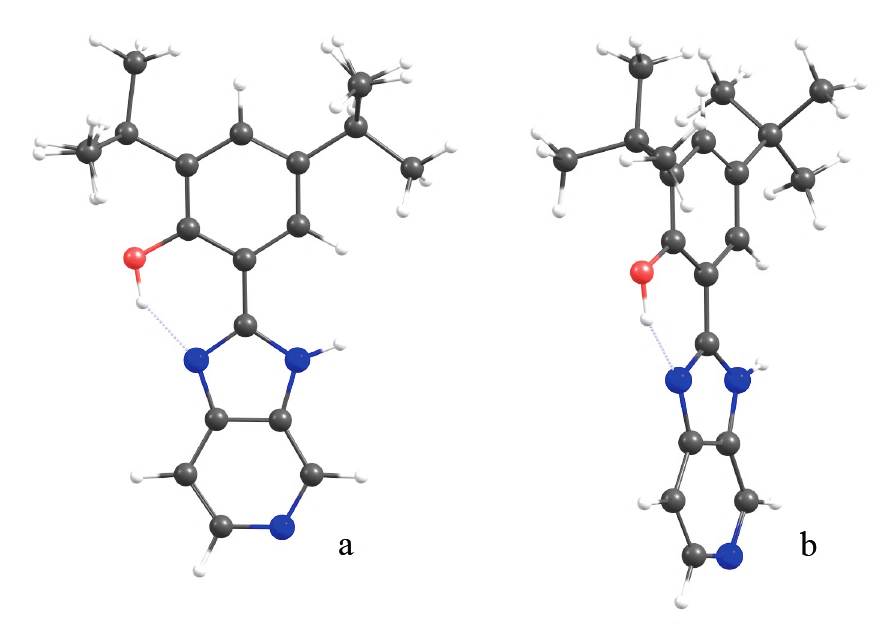

Supplement: Figure S1 — Molecular model of pyridine benzimidazole 2,4-di-tert-butyl-6-(3H-imidazo[4,5-c]pyridine-2-yl)phenol (B2) used in this study; front (A) and slide (B). [file Image_1.TIF]

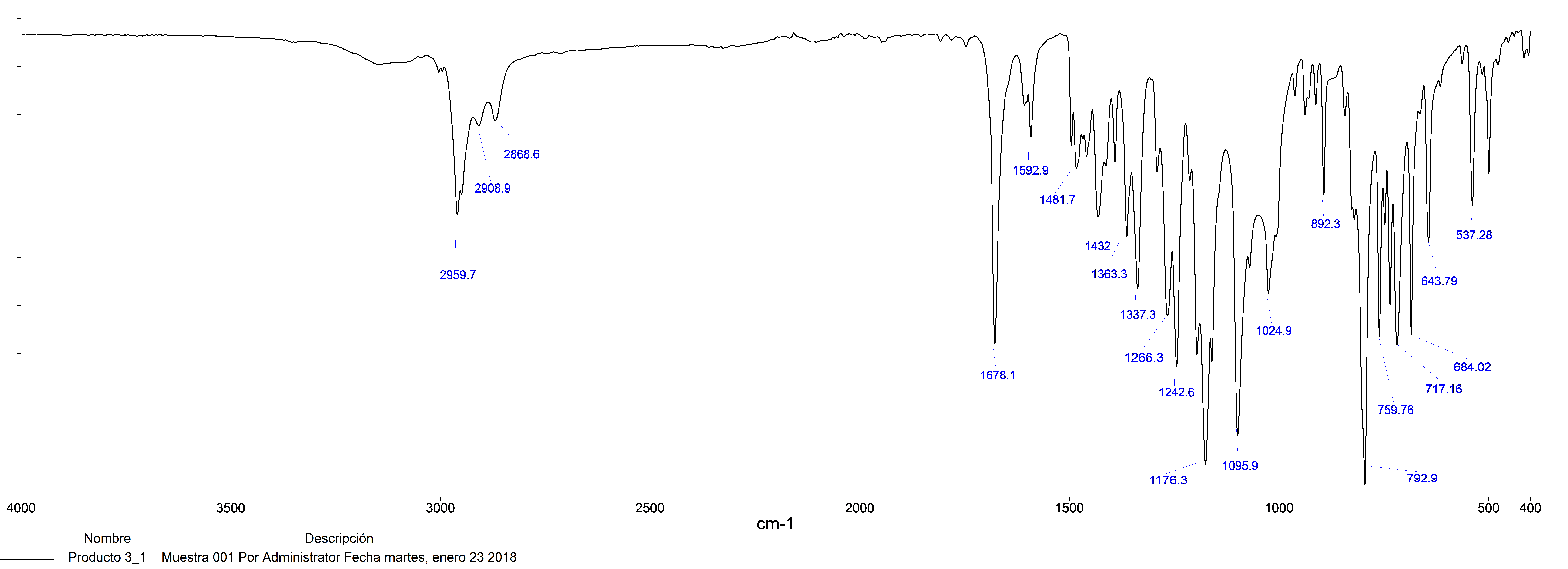

Supplement: Figure S2 — FTIR (ATR) of precursor (phenyl-3,5-di-tert-butyl-2-hydroxybenzoate). [file Image_2.TIF]

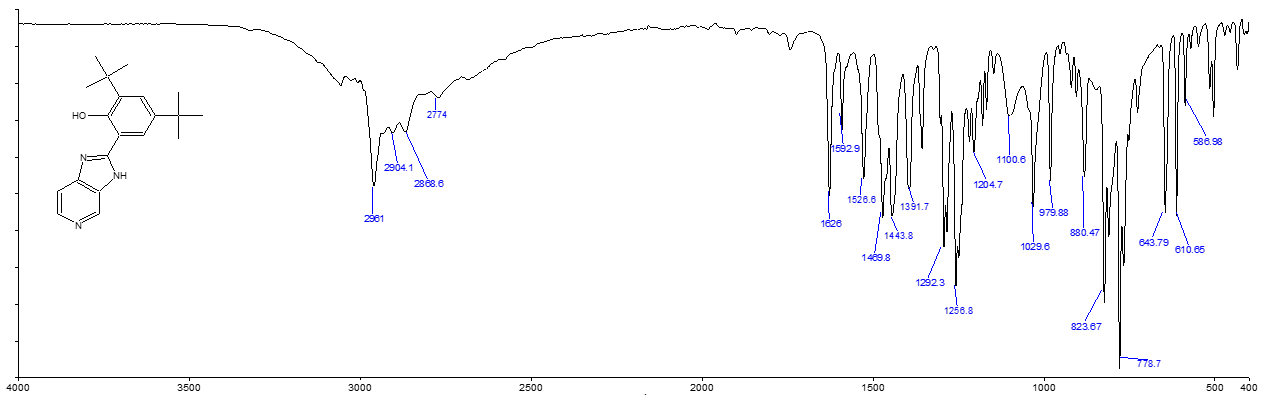

Supplement: Figure S3 — FTIR (ATR) of B2. [file Image_3.TIF]

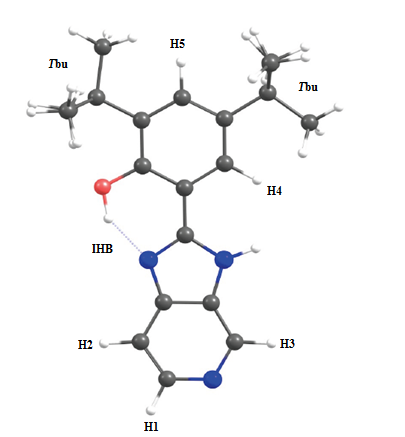

Supplement: Figure S4 — Numbering protons of B2. [file Image_4.TIF]

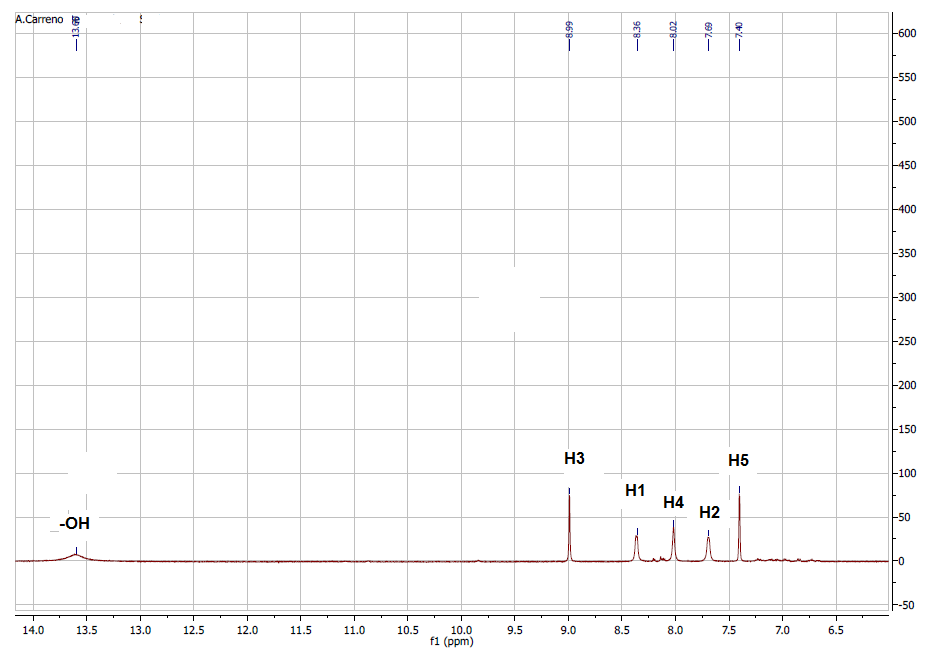

Supplement: Figure S5 — Aromatic zone of the 1HNMR spectrum of B2. [file Image_5.TIF]

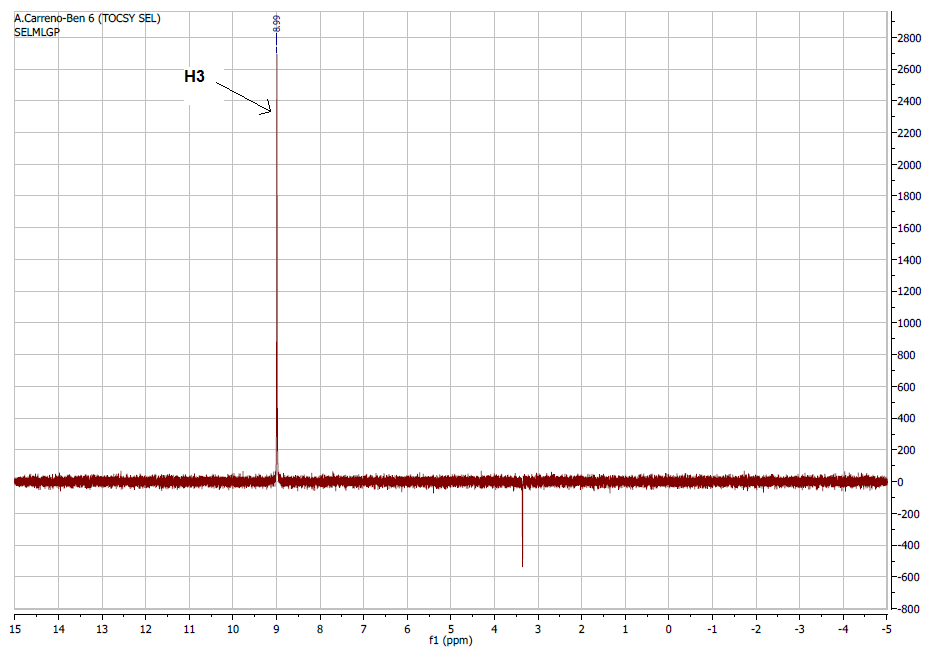

Supplement: Figure S6 — 1D TOCSY of B2 (in DMSO-d6), irradiating H3 at 3,597 Hz (8.99 ppm). [file Image_6.TIF]

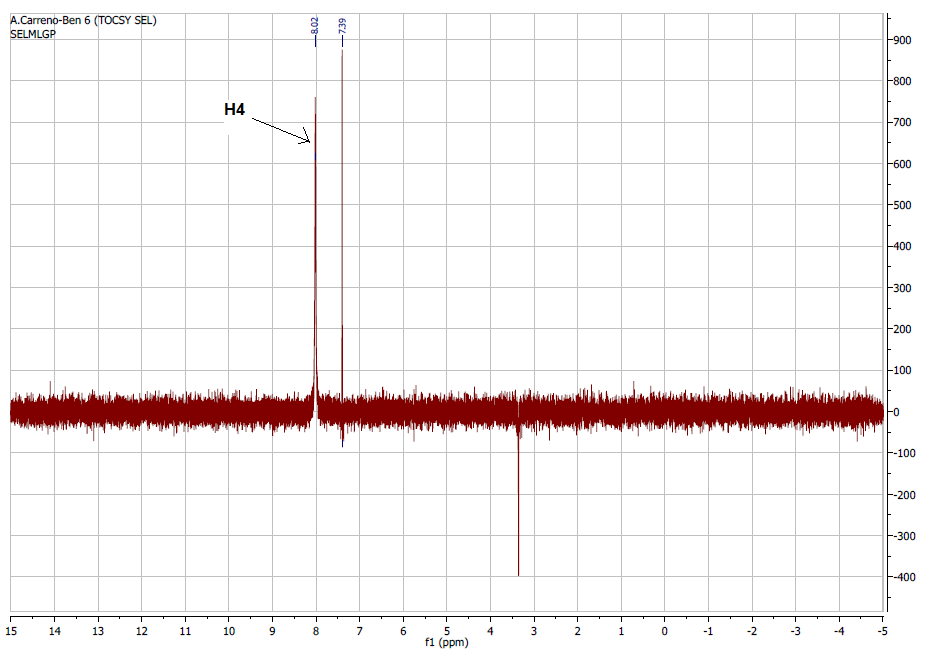

Supplement: Figure S7 — 1D TOCSY of B2 (in DMSO-d6), irradiating H4 at 3,208 Hz (8.02 ppm). [file Image_7.TIF]

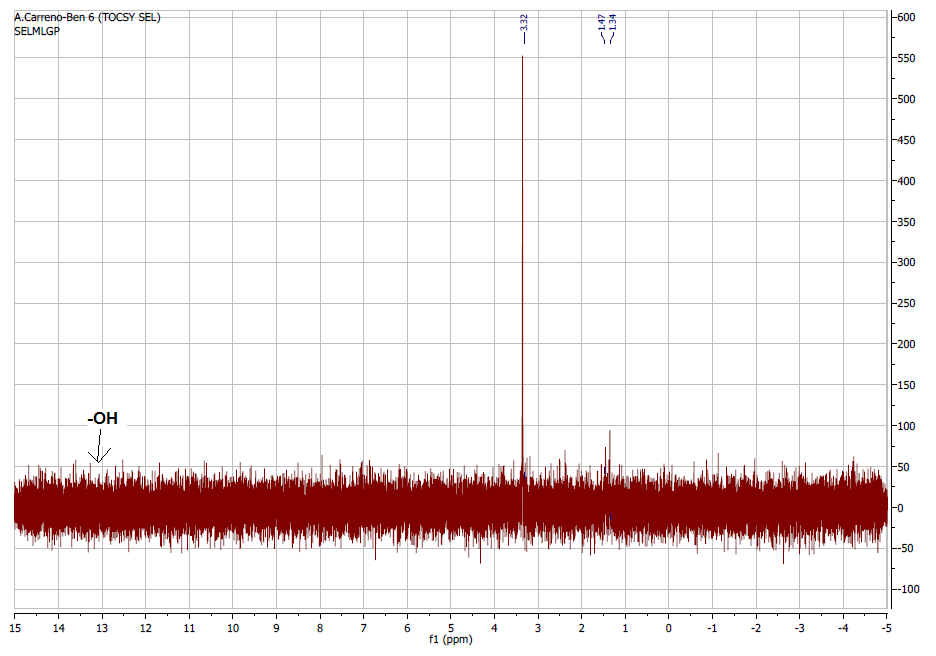

Supplement: Figure S8 — 1D TOCSY of B2 (in DMSO-d6), irradiating -OH at 5,451 Hz (13.62 ppm). [file Image_8.TIF]

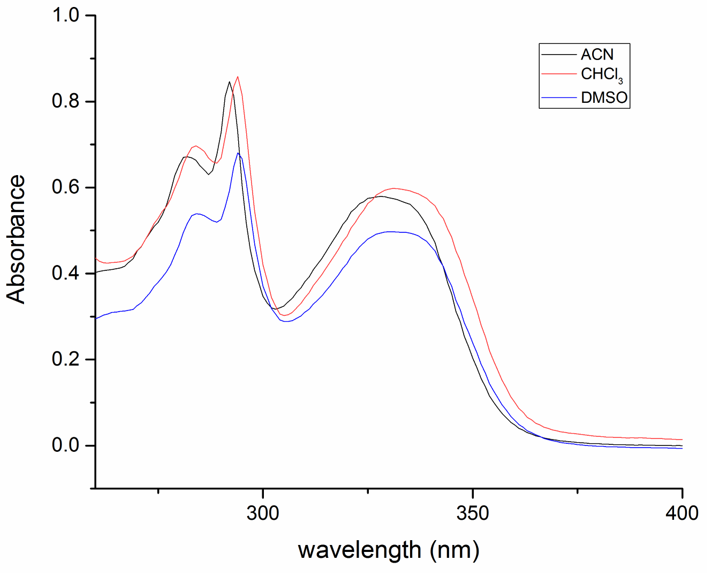

Supplement: Figure S9 — UV-Vis spectrum of B2 in chloroform, acetonitrile and DMSO at room temperature. [file Image_9.TIF]

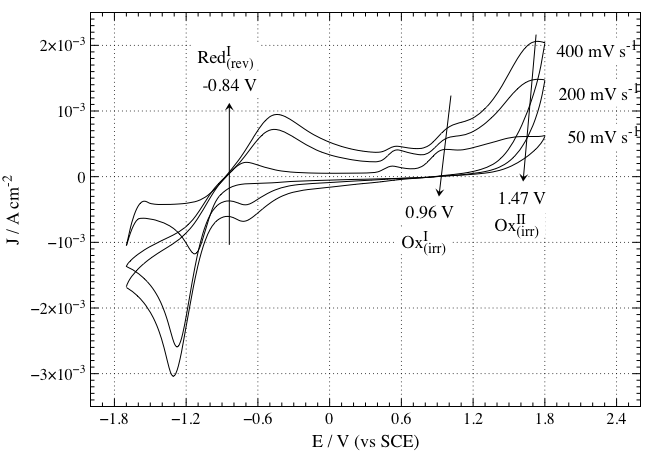

Supplement: Figure S10 — Scan-rate study for B2. Interphase: Pt|1.0·10−2 M B2 + 1.0·10−1 M TBAPF6 in anhydrous CH3CN. [file Image_10.TIF]
