# Supplementary material for: New Properties of a Bioinspired Pyridine Benzimidazole Compound as a Novel Differential Staining Agent for Endoplasmic Reticulum and Golgi Apparatus in Fluorescence Live Cell Imaging
Source: Front Chem. 2018 Aug 15;6:345. doi: 10.3389/fchem.2018.00345 (PMC6123694; doi:10.3389/fchem.2018.00345)
Supplement: Table S1 — Characteristic constants of phenyl-3,5-di-tert-butyl-2-hydroxybenzoate* and B2. [file Table_1.pdf]

SUPPLEMENTARY TABLES

Table S1. Characteristic constants of phenyl-3,5-di-tert-butyl-2-hydroxibenzoate* and **B1**.

**Compound**	**Molecular formula**	**MW (g mol^{-1})**	**Melting point (°C)**	**Solid color**	**TLC R_f**
**B2**	$C_{20}H_{25}N_3O$	**323,4320**	311-312	Light brown	0.38
phenyl-3,5-di-tert-butyl-2-hydroxibenzoate*	$C_{21}H_{26}N_3O_3$	**365,4256**	ND	White	0.80

MW: Molecular weight

TLC: Thin layer chromatography, using ethyl acetate as solvent

ND: not determined

* Precursor for **B2** synthesis

Table S2. UV-Vis absorption spectra of **B2** in different organic solvents at room temperature.

**Compound**	**Chloroform**		**Acetonitrile**		**DMSO**	
	**λ exp**	**ε**	**λ exp**	**ε**	**λ exp**	**ε**
**B2**	332<br>294<br>284	13.32<br>19.09<br>15.51	327<br>292<br>282	12.91<br>18.67<br>14.88	332<br>294<br>284	11.30<br>15.44<br>12.23

λ is in nm

ε is in $mol^{-1} dm^3 cm^{-1}$

Table S3. Scan-rate study for determining diffusional control of described electrochemical processes at **B2**.

**Process**	**Scan-rate vs current-density peak**	**(Scan-rate)^{½} vs current-density peak**	**Diff. control?**	**Process**	**Scan-rate vs current-density peak**	**(Scan-rate)^{½} vs current-density peak**	**Diff. control?**
	**m**	**n**	**R^2**	**m**	**n**	**R^2**	
$Red^{I}_{(rev)}$	-5.3×10^5	-2.2×10^2	0.833	-2.1×10^4	-3.2	0.921	yes
$Ox^{I}_{(irr)}$	9.5×10^5	3.6×10^2	0.989	3.5×10^4	-7.5	0.999	yes
$Ox^{II}_{(irr)}$	2.4×10^5	1.1×10^2	0.961	8.9×10^3	1.5	0.996	yes

"m", "n" and "R^2" are the slope, intercept and linear regression coefficient, respectively.

The scan-rate study provides a fast determination of the nature of electrochemical processes, allowing a differentiation between processes controlled by mass-transport (diffusion) from others. For each identified process for **B2**, several cyclic voltammetry runs must be recorded at different scan rates. Thus, two plots must be constructed, an "Scan-rate vs current-density peak" and a "(Scan-rate)^{½} vs current density peak" for each reduction an oxidation identified in the system (see **Table S3**).

Table S4. Results for the geometry optimization of the ground (S_0) and first excited singlet (S_1) state.

**Compound**	**d(N-H)**	**d(O-H)**
S_0	1.731	0.989
S_1	1.673	1.006

Table S5. The most important absorption and emission bands for **B2** in DMSO.

	**λ (nm)**	**f***	**Assignment**
**Absorption**	271.8	$1.1{\times}10^{-2}$	HOMO-2→LUMO+1 (n→π*)
	293.4	$1.2{\times}10^{-2}$	HOMO-1→LUMO (π→π*)
		$1.4{\times}10^{-1}$	HOMO→LUMO (π→π*)
**Emission**	514.8	$6.5{\times}10^{-3}$	LUMO→HOMO (π*→π)

*The oscillator strength was obtained using COSMO for DMSO as solvent.
